# The correlation between mitochondrial derived peptide (MDP) and metabolic states: a systematic review and meta-analysis

**DOI:** 10.1186/s13098-024-01405-w

**Published:** 2024-08-19

**Authors:** Qian Zhou, Shao Yin, Xingxing Lei, Yuting Tian, Dajun Lin, Li Wang, Qiu Chen

**Affiliations:** 1 Hospital of Chengdu University of Traditional Chinese Medicine, Sichuan, Chengdu, 610072 China; 2 Hospital of Chengdu University of Traditional Chinese Medicine, Sichuan Province, No. 39, Shi-Er-Qiao Road, Chengdu, 610072 People’s Republic of China

**Keywords:** Mots-c, Obesity, Diabetes, Meta-analysis, Mitochondrion

## Abstract

**Background:**

MOTS-c is known as mitochondrial open reading frame (ORF) of the twelve S c, produced by a small ORF-encoded peptides (SEPs) in mitochondrial 12S rRNA region. There is growing evidence that MOTS-c has a strong relationship with the expression of inflammation- and metabolism-associated genes and metabolic homeostasis, and even offering some protection against insulin resistance (IR). However, studies have reported inconsistent correlations between different population characteristics and MOTS-c levels. This meta-analysis aims to elucidate MOTS-c levels in physiological and pathological states, and its correlation with metabolic features in various physiological states.

**Methods:**

We conducted a systematic review and meta-analysis to synthesize the evidence of changes in blood MOTS-c concentration, and any association between MOTS-c and population characteristic. The Web of Science, PubMed, EMBASE, CNKI, WANGFANG and VIP databases were searched from inception to April 2023. The statistical analysis was summarized using the standardized mean difference (SMD) and 95% confidence interval (95% CIs). Pearson correlation coefficient was used to analyze the correlation and generate forest plots through a random-effects model. Additional analyses as sensitivity and subgroup analyses were performed to identify the origins of heterogeneity. Publication bias was retrieved by means of a funnel-plot analysis and Egger’s test. All related statistical analyses were performed using Revman 5.3 and Stata 15 statistical software.

**Result:**

There are 6 case–control studies and 1 cross-sectional study (11 groups) including 602 participants in our current meta-analysis. Overall analysis results showed plasma MOTS-c concentration in diabetes and obesity patients was significantly reduced (SMD = − 0.37; 95% CI− 0.53 to − 0.20; P < 0.05). After subgroup analysis, the present analysis has yielded opposite results for MOTS-c changes in obesity (SMD = 0.51; 95% CI 0.21 to 0.81; P < 0.05) and type 2 diabetes mellitus (T2DM) (SMD = − 0.89; 95% CI − 1.12 to − 0.65; P < 0.05) individuals. Moreover, the correlation analysis was performed to identify that MOTS-c levels were significantly positively correlated with TC (r = 0.29, 95% CI 0.20 to 0.38) and LDL-c (r = 0.30, 95% CI 0.22 to 0.39). The subgroup analysis results showed that MOTS-c decreased significantly in patients with diabetes (SMD = − 0.89; 95% CI− 1.12 to − 0.65; P < 0.05). In contrast, the analysis result for obesity persons (BMI > 28 kg/ m^2^) was statistically significant after overweight people (BMI = 24–28 kg/ m^2^) were excluded (SMD = 0.51; 95% CI 0.21 to 0.81; P < 0.05), which is completely different from that of diabetes. Publication bias was insignificant (Egger’s test: P = 0.722).

**Conclusion:**

Circulating MOTS-c level was significantly reduced in diabetic individuals but was increased significantly in obesity patients. The application of monitoring the circulating levels variability of MOTS-c in routine screening for obesity and diabetes is prospects and should be taken into consideration as an important index for the early prediction and prevention of metabolic syndrome in the future.

PROSPERO registration number CRD42021248167.

**Supplementary Information:**

The online version contains supplementary material available at 10.1186/s13098-024-01405-w.

## Introduction

The prevalence of metabolic diseases, including diabetes and obesity, is on the rise worldwide, which has amplified concerns about the health risks associated with this worsening health status [1, 2]. Obesity is a multifactorial inflammatory disease of maladaptive adipose tissue mass, typically associated with chronic insulin resistance (IR) [3]. Type 2 diabetes mellitus (T2DM) is a metabolic disease characterized by persistent hyperglycaemia secondary to insufficient insulin secretion and/or insulin resistance [4]. T2DM and related complication are increasingly recognized as important causes of mortality and morbidity worldwide, posing a major global health and economy threat [5]. Obesity individuals are accompanied by insulin resistance enhancing, hyperinsulinemia and risk of T2DM increasing. Subsequently, hyperglycemia can trigger dangerous medical complications, thereby aggravating vicious cycle and leading inexorably to worsening of obesity and T2DM [6]. Thus, an independent predictive biomarker at early stages of T2DM and obesity should be provided for early diagnosis and treatment in the daily clinical practice and large-scale clinical investigation.

Various interventions including nutritional interventions, lifestyle modification and increasing physical activity have been suggested to prevent and manage the symptoms of T2DM, but there is still no definitive treatment [7, 8]. Mitochondrial open-reading-frame (ORF) of the twelve S type-c (MOTS-c), a bioactive peptide involved in the regulation of metabolic homeostasis, is yielded by a small ORF-encoded peptides (SEPs) in mitochondrial 12S rRNA region [9]. There is growing evidence that MOTS-c has a strong relationship with the expression of inflammation- and metabolism-associated genes and plays an extensive impact in organismal and cellular metabolic homeostasis [10]. MOTS-c treatment could prevent high fat diet- or age-associated insulin resistance and diet-induced obesity in mice [9], and has drawn attention as a potential prevention or therapeutic option for diabetes and obesity [9, 11]. Treatment and overexpression of MOTS-c increased the AMP-activated protein kinase (AMPK) activity offering some protection against IR [12]. Thus, we speculate that MOTS-c has a protective effect in part population (especially obesity and diabetes) as a regulator for metabolic homeostasis.

Although research on the metabolic activity of MOTS-c is gradually increasing, there are several gaps in the correlation between population characteristics and MOTS-c levels reported in research reports. In addition, the key molecules and mechanisms of MOTS-c and mitochondrial related to metabolic regulation remain vague. This meta-analysis aims to elucidate MOTS-c levels in physiological and pathological states, and its correlation with metabolic features in various physiological states. The present meta-analysis demonstrates MOTS-c levels may serve as a sensitive and early indicator of the occurrence and development of obesity and diabetes.

## Methods

The present systematic review and meta-analysis was designed, conducted and reported based on the Preferred Reporting Items for Systematic Reviews and Meta Analysis (PRISMA) 2020 [13] guidance and Methodological Expectations of Cochrane Intervention Reviews (MECIR) [14] guidelines. The study was registered in the PROSPERO with the following registration number CRD42021248167.

### Date sources and search strategy

A systematic literature search was performed using the Web of Science, PubMed, EMBASE, CNKI, WANGFANG and VIP databases from inception to April 2023. The search used appropriate Medical Subject Headings and the use of following search terms based on PICO principle (Supplementary Table 1). We restricted the search to include only human studies, Chinese- or English-language publications, and full-text articles without time period limitations. Excluding irrelevant studies though reviewing the titles and/or abstracts, then two authors independently read the full texts of the remain studies. Relevant studies got qualified after joint review reaching agreement. In the several searches, searching strategy was combined two separate parts for obtaining a complete set of studies. In order to identify any missed papers, the lists of references of retrieved publications were also checked to identify additional relevant studies.

### Study selection and exclusion criteria

Clinical trials were identified which fulfil the following criteria will be included: (1) original studies published in Chinese- or English-language, peer-reviewed journals; (2) restricted the search to include only human studies; (3) participants had a history of confirmed diabetes or obesity diagnosis. Clinical trials with the following characteristics were excluded: (1) individuals with any accompany disease, including psychiatric disorders, stroke, cancer, renal disease or severe hepatic, and acute cardiovascular events, et al.; (2) meta-analysis, reviews, meeting abstracts, comments and letters, and posters; and (3) the unpublished articles or non-research articles were excluded.

### Data extraction and quality assessment

Data from included studies were extracted by two authors (XL, SY) independently according to a predefined standardized format. The extracted items as follows: study basic information (first author’s name, published year, location, sample size, etc.); and included participant characteristics (body mass index (BMI), age, MOTS-c level, disease type, homeostatic model assessment of insulin resistance (HOMA-IR), Total Cholesterol, and correlation coefficients between metabolic characteristics and MOTS-c). For quality assessment of included studies, using Newcastle–Ottawa Scale (NOS) adapted for case–control and cross-sectional studies [15]. Any discrepancy or ambiguity in Data extraction process and quality assessment between the two researchers was resolved by consultation with a third researcher until a consensus was reached (QZ).

### Data synthesis and analysis

For the statistical analysis, Standard Mean Difference (SMD) with 95% confidence interval (CI) for continuous outcomes and Risk Ratio (RR) with 95% CI for dichotomous outcomes were used to estimate the pooled effects. We estimated the associations between different metabolic features and MOTS-c levels using Pearson correlation coefficients and generated forest plots through a random-effects model. Correlation coefficients were normalized to z values via Fisher’s z-transformation to calculate the relevant statistics. Meta-analyses produced variance and 95% CI before translating them back to the summary effect size (r). Heterogeneity was tested though Cochrans Q statistic and the proportion of the total variation resulted from heterogeneity was quantified via the I^2^ statistic [16], when I^2^ > 50% and P < 0.05 were considered to indicate significant heterogeneity [17]. Additional analyses as sensitivity and subgroup analyses were performed to identify the origins of heterogeneity. Publication bias was retrieved by means of a funnel-plot analysis, and the Egger’s test between included studies and P < 0.05 were considered to indicate statistically significant [18]. All related statistical analyses were performed using Revman 5.3 and Stata 15 statistical software.

## Results

### Literature search results

**Fig. 1:**
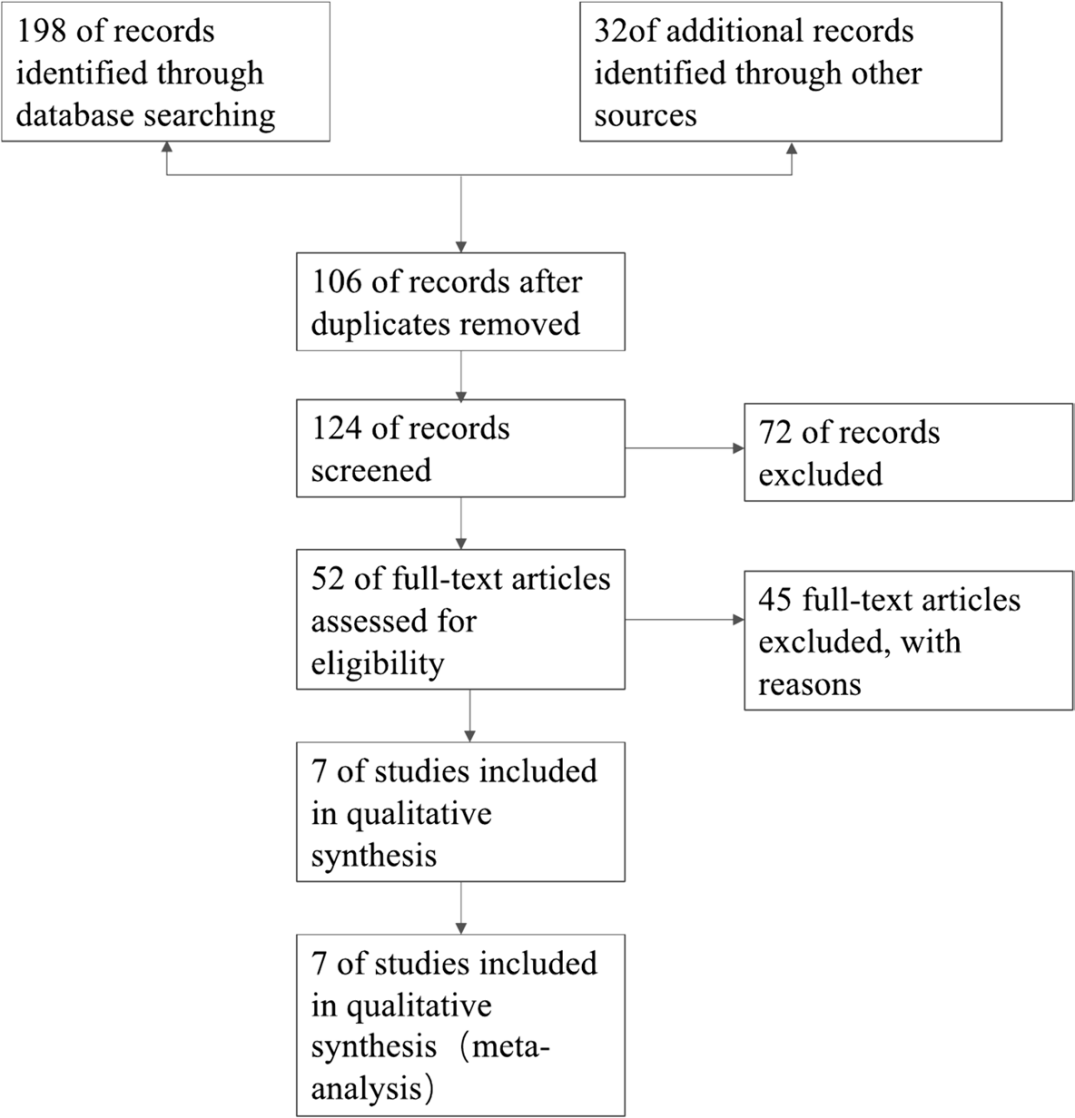
Flow chart of literature search

The flow chart demonstrating the selection process with more details is shown in Fig. 1. Through electronic database search, 198 citations were initially identified, including PubMed, Embase, Web of Science, CNKI, WANGFANG and VIP databases. Due to duplicate papers, review, and non-human, 106 studies were eliminated. The title and abstract of each article were examined, and 72 ineligibles titles were removed. 45 articles were excluded after reading the full texts. Finally, 7 studies (Baylan, F. A. [19]; Du, C. [20]; Ramanjaneya, M. [21]; Cataldo, L. R. [22]; Jiang Fen [23]; Wojciechowska M. [24]; Wang Xiaogang [25]) were included in this meta- analysis. Features of the 7 included studies between 2018 and 2022, 5 were published in English, and 2 were published in Chinese. Out of them, 6 included individuals with Obesity, 3 included individuals with T2DM. In Ramanjaneyas’s [21] study, the subjects were divided into two groups as T2DM with HbA1c < 7% and T2D with HbA1c > 7%. In Cataldo's [22] study, the subjects were divided into Males groups and Females groups. In Jiang Fen [23] study, participants were split into three groups(T2DM, Obesity with BMI = 24–28 and Obesity with BMI > 28). Thus, from inception to 2023, there are 7 published studies with 11 groups, and 661 participants were selected in our present meta-analysis. The authors estimated all eligible studies clinical information though anthropometric measurements. Summing up the detailed characteristics of selected studies in Table 1, and the sample size ranged from 5 to 93.

**Table 1:** Clinical and metabolic features of included studies

Authors	Publication year	Country	Female/male	Study design	Follow-up period	Underling diseases	Sample size	Circulating MOTS-c (mean ± SD)		Age (y)
								Mean	SD	
Baylan, F. A. [19]	2021	Turkey	Not available	Case control	Not available	Normal	14	211.4	119.7	50.4 ± 12.6
						Obese (BMI > 28)	18	190.4	115.4	56.4 ± 5.9
Du, C. [20]	2018	China	17/40	Case control	2017	Normal	57	561.64	19.19	9.55 ± 0.33
			13/27			Obese (BMI = 24–28)	40	472.61	22.8	10.37 ± 0.33
Ramanjaneya, M. [21]	2019	Qatar	35/33	A cross-sectional study	Not available	Normal	68	235.3	181.6	49.3 ± 10.6
			18/13			T2DM (HbA1c < 7%)	31	186.3	125.6	54.7 ± 11.2
			37/56			T2DM (HbA1c > 7%)	93	157.7	136.6	57.2 ± 8.3
Cataldo, L. R. [22]	2018	Chile	/	Case control	Not available	Normal males	5	0.54	0.21	35.2 ± 8.5
						Obese males (BMI > 28)	5	0.49	0.11	37.8 ± 3.1
						Normal females	5	0.42	0.08	31.1 ± 5.0
						Obese females (BMI > 28)	5	0.55	0.19	34.0 ± 10.6
Jiang Fen [23]	2020	China	28/15	Case control	2018–2019	Normal	43	72.36	19.78	53.36 ± 10.01
			39/27			T2DM	66	12.88	11.4	44 ± 15.01
			Not available			Normal	66	44.51	28.18	Not available
						Obese (BMI = 24–28)	40	66.44	28.29	Not available
						Obese (BMI > 28)	43	54.49	35.68	Not available
Wojciechowska M [24]	2021	Poland	9/9	Case control	Not available	Normal	18	427.66	148.93	30.09 ± 1.300
			12/10			Obese (BMI > 28)	22	976	204.84	32.62 ± 1.243
Wang Xiaogang [25]	2022	China	/	Case control	2019–2021	Normal	Not available			
			12/10			T2DM	22	234.22	98.31	58.33 ± 4.23

### Overall analysis

**Fig. 2:**
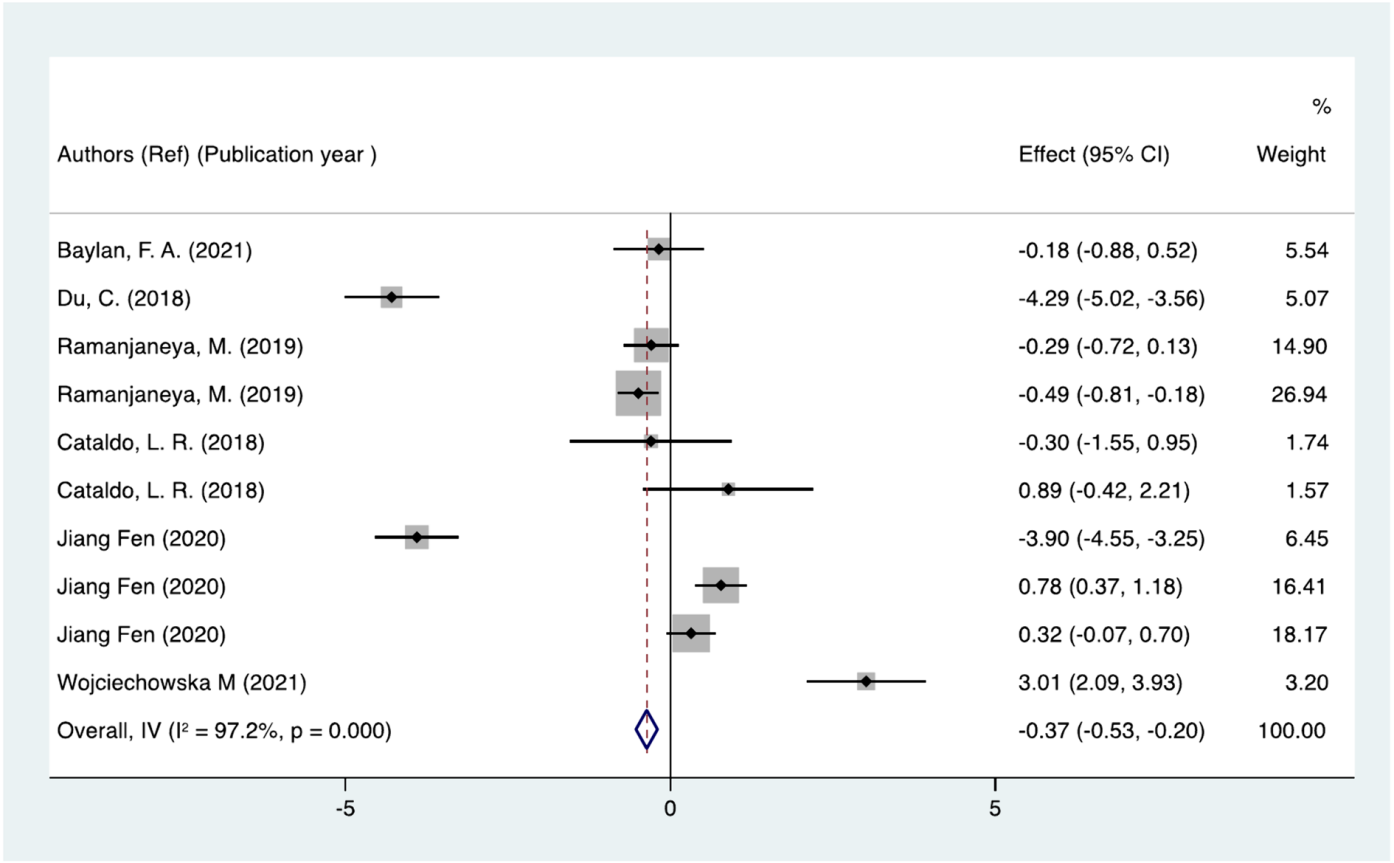
Overall analysis results. CI, Confidence interval. Summary estimates were analyzed using a random-effects model

The analysis results demonstrate that plasma MOTS-c concentration is significantly reduced in all included individuals as shown in Fig. 2 (SMD = − 0.37; 95% CI: − 0.53 to − 0.20; P < 0.05) with substantial heterogeneity by a random effect model (I^2^ = 97.2%, P = 0.000). As showed in Supplementary Fig. 1, MOTS-c levels were significantly positively correlated with Total Cholesterol (TC) (r = 0.29, 95% CI 0.20 to 0.38) and Low-Density-Lipoprotein cholesterol (LDL-c) (r = 0.30, 95% CI 0.22 to 0.39). The analysis results showed insignificant heterogeneity by a random effect model for TC (I^2^ = 0.0%, P = 0.693) and significant heterogeneity for LDL-c (I^2^ = 85%, P < 0.05). However, no significant correlation was found for other indicators (P > 0.05), such as BMI, HOMA-IR and Age. In order to determine the cause of heterogeneity, we have thus performed the necessary analyses below.

### Subgroup and sensitivity analyses

**Fig. 3:**
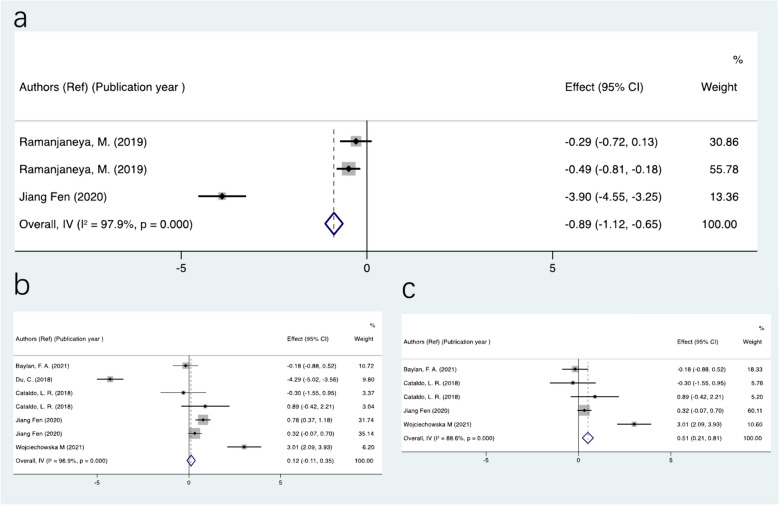
The SMDs of MOTS-c concentration depended on disease types and severity of symptoms. a) diabetes; b) obesity included overweight people (BMI = 24–28 kg/m^2^); c) obesity (BMI > 25 kg/m^2^) excluded overweight people

Subgroup and Sensitivity analyses were performed to find the sources of heterogeneity. Since all subjects in the research reported obesity or diabetes, we speculated that heterogeneity was related to the disease types, severity and profile of symptoms. The various data analyses for T2MD and Obesity subgroups yielded varying results, which are presented in Fig. 3. The results showed that MOTS-c decreased significantly in patients with diabetes (SMD = − 0.89; 95% CI − 1.12 to − 0.65; P < 0.05), similar to what was previously found (Fig. 2). In contrast, the analysis result for obesity persons (BMI > 28 kg/ m^2^) was statistically significant after overweight people (BMI = 24–28 kg/ m^2^) were excluded (SMD = 0.51; 95% CI 0.21 to 0.81; P < 0.05), which is completely different from that of diabetes. In current meta-analysis, subgroup analyses regarding several other factors that could impact the association failed to be completed due to the under-representation number of trials in correlation analysis. After subgroup analysis, we discovered that heterogeneity was remained considerably high when compared to previous studies. We therefore performed further sensitivity analyses for each end point by excluding individual studies. The results of the sensitivity-pooled SMD on the bulk of the outcomes indicated that all exclusions had no effect on the prior analyses results.

### Publication bias and quality assessment

Symmetrical dispersion points (Supplementary Fig. 2) and the Egger test were used to assess the presence of potential publication bias. Test confirmed that publication bias was evaluated and considered insignificant (Egger's test: P = 0.722; Supplementary Fig. 3). The Newcastle–Ottawa Scale and common excel files were used to evaluate the methodological quality and bias of all qualifying studies. The quality of included studies was assessed by NOS quality assessment scale with a score ranging from five to eight stars (Tables 2–3).

**Table 2:** Quality Assessment of Studies Using Newcastle–Ottawa Scale for Case–control Studies

Authors, year	Selection				Comparability of cases and controls (matched for)	Exposure			Total score
	Adequate definition of cases	Representativeness of cases	Selection of controls	Definition of controls		Ascertainment of exposure	Same method of ascertainment for cases and controls	Non-response rate	
Baylan, F. A., [19]	*	*	–	*	**	*	*	–	7
Du, C., [20]	*	*	*	*	**	*	*	–	8
Cataldo, L. R., [22]	*	*	–	*	**	*	*	*	8
Jiang Fen, [23]	*	*	–	*	**	–	*	–	6
Wojciechowska M, [24]	*	*	*	*	**	*	*	–	8
Wang Xiaogang, [25]	*	*	–	*	**	*	*	–	7

**Table 3:** Newcastle–Ottawa Scale, adapted for quality assessment of cross-sectional studies

Authors, year	Selection				Comparability	Assessment of the outcome		Quality rating
	Representativeness of the sample	Sample size	Non-respondents	Ascertainment of the exposure	Based on design and analysis	Assessment of the outcome	Same method of ascertainment for cases and controls	
Ramanjaneya, M., [21]	–	–	–	*	**	*	*	5

## Discussion

To our knowledge, this was the first meta-analysis to elucidate the blood concentration changes of MOTS-c peptide and its correlation with different metabolic features in various physiological states. The present analysis has yielded opposite results for plasma MOTS-c concentration changes in obesity (significantly increased) and diabetic (significantly decreased) individuals. Results from correlation analyses revealed that MOTS-c was positively associated with TC and LDL-c. This connection result is in line with prior analysis results of Most-c increased significantly in obesity individuals. However, no correlation was observed for other measures of obesity, which could be explained by the paucity of literature reporting pertinent data. Data provides evidence that MOTS-c may be a new therapeutic target for obesity and diabetes. And it may be useful to predict metabolic syndrome by monitoring the level of MOTS-c.

According to our analysis results, several studies reached a similar conclusion, as MOT-c expression were lower in T2MD and related to the hemoglobin [22]. For obesity, there are different views. Insufficient sample size, varied assay method, diverse detected sample and different characteristics existing in study designs may underlie discrepancies among existing bodies of evidence. Cataldo. L. R [22] suggested that plasma MOTS-c level depends on the metabolic status, and MOTS-c concentration associates positively with insulin resistance in lean individuals. Lu, H [26] suggested that MOTS-c is a high potential candidate for chronic treatment of menopausal induced metabolic dysfunction. MOTS-c peptide regulates adipose homeostasis to prevent ovariectomy-induced metabolic dysfunction [26]. Kim, S. J [12] found that three pathways were reduced in MOTS-c–injected mice, including sphingolipid metabolism, monoacylglycerol metabolism, and dicarboxylate metabolism. And these pathways are upregulated in obesity and T2DM models. During obesity, generated oxidative stress contributes to the formation of peroxynitrite, which increases the production of reactive oxygen species (ROS) and promotes cytochrome C-related damage in the mitochondrial electron transfer chain [27]. Above representative metabolites were strongly associated with the risk of developing T2MD and obesity. Therefore, as chronic diseases, early detection play an essential role in diagnosis, treatment, and comprehensive care of patients.

Mitochondrially derived peptides as novel regulators of metabolism. And mitochondrial-derived peptides (MDPs) have also been found to affect metabolism. These MDPs have profound and distinct biological activities, and provide a paradigm-shifting concept of active mitochondrial-encoded signals that act at the cellular and organismal level (i.e. mitochondrial hormone) [28, 29]. Lee C and Zeng J et al. [9] have suggested a hypothesis that mitochondria may actively regulate metabolic homeostasis at the cellular and organismal level via peptides encoded within their genome. In investigations on mice, MOTS-c has been shown to be a mitochondrial-derived peptide that targets the skeletal muscle and enhances glycolipid metabolism [30], effectively preventing high-fat diet-induced insulin resistance and obesity as well as age-dependent insulin resistance [9]. Lee C and Kim KH et al. [30] hypothesized MOTS-c actions in vivo would be related to insulin sensitivity and glucose handling, as it enhanced glucose flux rate in vitro and acute-treatment reduced glucose levels by regulating the cellular entry and utilization of glucose in mice fed a normal diet. The action of MOTS-c represents an entirely novel mitochondrial signaling mechanism. Guo Q [31] suggested that treated with adiponectin in mice regulating the expression of the mitochondrial-derived peptide MOTS-c, and its response to improves insulin resistance via APPL1-SIRT1-PGC-1α. Similar results were obtained by Yang B [32], MOTS-c interacts synergistically with exercise intervention to regulate PGC-1α expression, attenuating insulin resistance and enhance glucose metabolism in mice via AMPK signaling pathway. Kong BS [33] has found that MOTS-c prevents pancreatic islet destruction in autoimmune diabetes. Additional, Sequeira IR [34] has found a significant association between visceral fat mass and plasma MOTS-c.

In the current meta-analysis, no statistically significant changes were observed for MOTS-c in obesity population while overweight participants were included, but it significantly increased since they were eliminated. For diabetic individuals, the plasma MOTS-c concentration showed dramatically decreased, which was opposite expression compared with obesity. According to statistics, T2MD is a major complication of obesity [35]. And in the three subjects of T2MD included in the meta-analysis, all participants accompanied by an obesity phenotype. Therefore, we speculate that MOTS-c secretion will increase in the early metabolic imbalance of the obesity population, and decrease when obesity induced diabetes, which could possibly be related to an increase in hemoglobin. The results give additional evidence that mitochondrial dysfunction contributes to the development of diabetes development. Thus, we speculate MOTS-c may be considered as a potential monitoring indicator and therapeutic direction for obesity and diabetes based on the modulation of mitochondrial biogenesis. Due to the limited researches that is currently available, this interpretation may be valid only for obesity induced diabetes and fail to find other correlations. We definitely require further clinical data to support our conclusions since the results cannot accurately reflect the outcomes of clinical studies.

## Limitation

This meta-analysis has several inescapable limitations that need to be taken into further account consideration. Firstly, there was high heterogeneity among the controlled trials included in the analysis. Secondly, the language is restricted to Chinese and English, which introduces selection bias. Thirdly, further subgroup analysis was not allowed for correlation analysis, because the sample size was not sufficient. Lastly, the results were inconclusive because of the number of articles that were eligible for inclusion was limited. Therefore, there is an urgent need for further trials in reality. Despite the above-mentioned limitations, this mete-analysis and systematic review nonetheless offer insightful information.

## Conclusion

In summary, these existing experimental results support our speculation. As such, MOTS-c has implications in the regulation of obesity and diabetes. Application of monitoring MOTS-c in routine obesity and diabetes screening is possible, and should be taken into consideration for prediction and prevention of metabolic syndrome in an early stage. Despite some limitations in our study, we believe that this meta-analysis has significance for follow-up research to explore the possible pathophysiological mechanisms underlying this relationship. Additional studies are required to determine the role of MDPs in the metabolic dysregulation within and between cells of metabolic syndrome. As a crucial tool in the future battle against metabolic disorders. In this regard, the development of drugs aimed at the regulation of these processes is gaining attention.

### Supplementary Information

Supplementary Material 1. Figure 1: Associations between different metabolic features and MOTS-c using Pearson correlation coefficients. a) age; b) BMI; c) HOMA-IR; d) LDL-c; e) TC.

Supplementary Material 2. Figure 2: Funnel plot for publication bias analysis of the selected studies.

Supplementary Material 3. Figure 3: The result of Egger’s test.

Supplementary Material 4.

## Data Availability

On request, data were extracted from original research and data used in meta-analyses are accessible.

## Abbreviations

MOTS-c: Mitochondrial open reading frame (ORF) of the twelve S c
IR: Insulin resistance
SMD: Standard mean difference
CI: Confidence interval
T2DM: Type 2 diabetes mellitus
ORF: Open-reading-frame
SEPs: ORF-encoded peptides
AMPK: AMP-activated protein kinase
BMI: Body mass index
HOMA-IR: Homeostatic model assessment of insulin resistance
ROS: Reactive oxygen species
MDPs: Mitochondrial-derived peptides
PRISMA: Preferred reporting items for systematic reviews meta-analyses
RCTs: Randomized controlled trial
