# Supplementary material for: The correlation between mitochondrial derived peptide (MDP) and metabolic states: a systematic review and meta-analysis
Source: Diabetol Metab Syndr. 2024 Aug 19;16:200. doi: 10.1186/s13098-024-01405-w (PMC11331736; doi:10.1186/s13098-024-01405-w)
Supplement: Supplementary file 1 — Supplementary Material 1. Figure 1: Associations between different metabolic features and MOTS-c using Pearson correlation coefficients. a) age; b) BMI; c) HOMA-IR; d) LDL-c; e) TC. [file 13098_2024_1405_MOESM1_ESM.docx]

Supplementary Figure 1. Associations between different metabolic features and MOTS-c using Pearson correlation coefficients. a) age; b) BMI; c) HOMA-IR; d) LDL-c; e) TC.

BMI, body mass index; HOMA-IR, homeostatic model assessment of insulin resistance; TC, Total cholesterol; LDL-c, low density lipoprotein cholesterol.
