# Supplementary material for: The correlation between mitochondrial derived peptide (MDP) and metabolic states: a systematic review and meta-analysis
Source: Diabetol Metab Syndr. 2024 Aug 19;16:200. doi: 10.1186/s13098-024-01405-w (PMC11331736; doi:10.1186/s13098-024-01405-w)
Supplement: Supplementary file 2 — Supplementary Material 2. Figure 2: Funnel plot for publication bias analysis of the selected studies. [file 13098_2024_1405_MOESM2_ESM.docx]

Supplementary Figure 2. Funnel plot for publication bias analysis of the selected studies. 
