# Supplementary material for: The correlation between mitochondrial derived peptide (MDP) and metabolic states: a systematic review and meta-analysis
Source: Diabetol Metab Syndr. 2024 Aug 19;16:200. doi: 10.1186/s13098-024-01405-w (PMC11331736; doi:10.1186/s13098-024-01405-w)
Supplement: Supplementary file 3 — Supplementary Material 3. Figure 3: The result of Egger’s test. [file 13098_2024_1405_MOESM3_ESM.docx]

Supplementary Figure 3. The result of Egger's test.
