# Supplementary material for: The correlation between mitochondrial derived peptide (MDP) and metabolic states: a systematic review and meta-analysis
Source: Diabetol Metab Syndr. 2024 Aug 19;16:200. doi: 10.1186/s13098-024-01405-w (PMC11331736; doi:10.1186/s13098-024-01405-w)
Supplement: Supplementary file 4 — Supplementary Material 4. [file 13098_2024_1405_MOESM4_ESM.docx]

**Supplementary table1. Keywords used for the search strategy.**

| **Population** | **Intervention** | **Control** | **Outcomes** |
| --- | --- | --- | --- |
| Diabetes Mellitus,  Type 1 Diabetes Mellitus,  T1DM,  T2DM,  Type 2 Diabetes Mellitus,  Diabetes Mellitus,  Diabetes Gestational,  Prediabetic State,  Overweight  Obesity  Obese | Insulin resistance,  Body Mass Index,  BMI,  Body Weights,  HOMA-IR,  Lipids,  Lipid,  Fats, | control group, cohort,  prospective, retrospective， follow-up，randomized control trial，case-control | MOTS-c,  mitochondrial open reading frame of the 12S rRNA-c,  Mitochondrial Proteins,  MOTS-c peptide, |
